# The significance of glucosinolates for sulfur storage in Brassicaceae seedlings

**DOI:** 10.3389/fpls.2014.00704

**Published:** 2014-12-19

**Authors:** Tahereh Aghajanzadeh, Malcolm J. Hawkesford, Luit J. De Kok

**Affiliations:** ^1^Laboratory of Plant Physiology, University of GroningenGroningen, Netherlands; ^2^Plant Biology and Crop Science Department, Rothamsted ResearchHarpenden, UK

**Keywords:** Brassicaceae, glucosinolate, hydrogen sulfide, myrosinase activity and expression, sulfur deficiency, sulfur dioxide, sulfur storage

## Abstract

*Brassica juncea* seedlings contained a twofold higher glucosinolate content than *B. rapa* and these secondary sulfur compounds accounted for up to 30% of the organic sulfur fraction. The glucosinolate content was not affected by H_2_S and SO_2_ exposure, demonstrating that these sulfur compounds did not form a sink for excessive atmospheric supplied sulfur. Upon sulfate deprivation, the foliarly absorbed H_2_S and SO_2_ replaced sulfate as the sulfur source for growth of *B. juncea* and *B. rapa* seedlings. The glucosinolate content was decreased in sulfate-deprived plants, though its proportion of organic sulfur fraction was higher than that of sulfate-sufficient plants, both in absence and presence of H_2_S and SO_2_. The significance of myrosinase in the *in situ* turnover in these secondary sulfur compounds needs to be questioned, since there was no direct co-regulation between the content of glucosinolates and the transcript level and activity of myrosinase. Evidently, glucosinolates cannot be considered as sulfur storage compounds upon exposure to excessive atmospheric sulfur and are unlikely to be involved in the re-distribution of sulfur in *B. juncea* and *B. rapa* seedlings upon sulfate deprivation.

## INTRODUCTION

Glucosinolates are secondary sulfur compounds commonly found in relatively high levels in shoots, roots, and seeds of Brassicaceae and may account for up to 20% of the organic sulfur fraction (Schnug, 1990, 1993; Fahey et al., 2001; Castro et al., 2004; Halkier and Gershenzon, 2006; Clay et al., 2009; Del Carmen Martinez-Ballesta et al., 2013). These secondary sulfur compounds are responsible for the spicy flavor of many species of the Brassicaceae, e.g., mustard and radish (Schnug, 1990, 1993). Glucosinolates are derived from amino acids and have a core structure consisting of a β-D-glucopyranose residue linked to a (*Z*)-*N*-hydroximino sulfate ester via a sulfur atom and a variable side chain (Halkier and Gershenzon, 2006). On the basis of their precursor amino acids, specific aliphatic, indolyl, and aromatic glucosinolates can be distinguished, and furthermore *Brassica* species differ strongly in content and composition of these glucosinolates (Castro et al., 2004; Halkier and Gershenzon, 2006).

The glucosinolate content may be affected by the sulfur nutritional status of the plant; supplemental sulfur fertilization of Brassica in greenhouse and field experiments resulted in an up to a 20-fold increase in glucosinolate content in foliar tissues (Falk et al., 2007). However, the impact of sulfur fertilization on glucosinolate content varied strongly between plant species, growth stage and organs, and the rate of sulfur supplied (Kirkegaard and Sarwar, 1998; Castro et al., 2004; Falk et al., 2007; Antonious et al., 2009). For instance, in some cultivars of broccoli, sulfur fertilization only resulted in an increase in glucosinolate content in the heads, whereas in other cultivars it had no effect or even resulted a lower glucosinolate content (Falk et al., 2007).

Upon cellular injury, glucosinolates are enzymatically degraded by myrosinase (a thioglucosidase), resulting in a variety of breakdown products, including glucose, sulfate, and depending on specific chemical structure, isothiocyanates, nitriles, epithionitriles, oxazolidinethions, indolyl alcohols, thiocyanate, and amines (Fenwick et al., 1983; Halkier and Du, 1997; Bones and Rossiter, 2006; Halkier and Gershenzon, 2006; Kissen and Bones, 2009; Kissen et al., 2009; Ahuja et al., 2010). It is presumed that both glucosinolates and their breakdown products may play a role in the defense of plants against microorganisms, fungi, and insects (Ernst, 1993; Bones and Rossiter, 1996; Ahuja et al., 2010). Moreover, glucosinolates are presumed to have a sulfur storage role in plants and their degradation by myrosinase might have significance in the re-distribution of sulfur in plants under sulfur-deprived conditions (Schnug, 1990; Hirai et al., 2004, 2005; Bloem et al., 2007; Falk et al., 2007).

In addition to sulfate taken up by the roots, plants are able to utilize foliarly absorbed sulfur gases as a supplemental sulfur source for growth (De Kok et al., 2000a, 2007, 2009). For instance, continuous exposure of Brassica to atmospheric H_2_S or SO_2_ levels of ≥0.2 μl l^-1^ was sufficient to cover the organic sulfur requirement to maintain growth in the absence of sulfate in the root environment (De Kok et al., 2000b, 2002; Yang et al., 2006a,b; Koralewska et al., 2008). In the current study, the impacts of sulfur nutrition (atmospheric and pedospheric) on the glucosinolate content and the transcript levels and activity of myrosinase were studied in seedlings of two Brassica species, which are characterized by a high (*Brassica juncea*, mustard greens) and low (*B. rapa*, mustard spinach) glucosinolate content. The aim of the study was to gain insight into the significance of glucosinolates in sulfur storage and the role of myrosinase in the re-distribution of sulfur in sulfate-deprived plants.

## MATERIALS AND METHODS

### PLANT MATERIAL AND H_2_S AND SO_2_ EXPOSURE

Seeds of *B. juncea* cv. Rugosa and *B. rapa* cv. Komatsuna; Van der Wal, Hoogeveen, The Netherlands) were germinated in vermiculite in a climate-controlled room. Day and night temperatures were 22 and 18°C (±1°C), respectively, relative humidity was 60–70%. The photoperiod was 14 h at a photon fluence rate of 300 ± 20 μmol m^-2^ s^-1^ (400–700 nm) at plant height, supplied by Philips GreenPower LED (deep red/white 120) production modules. Ten day-old seedlings were transferred to an aerated 25% Hoagland nutrient solution at 0.5 mM sulfate for 3 days and subsequently transferred to fresh Hoagland nutrient solution at 0 mM sulfate (-S, sulfate-deprived) or 0.5 mM sulfate (+S, sulfate-sufficient) in 13 l stainless steel containers (10 sets of plants per container; three plants per set). Plants were exposed to 0.25 μl l^-1^ H_2_S or SO_2_ for 7 days in 150 l cylindrical stainless steel cabinets (0.6 m diameter) with a polymethyl methacrylate top. Sealing of the lid of the container and plant sets prevented absorption of atmospheric H_2_S or SO_2_ by the solution. Day and night temperatures were 24 and 20°C (±2°C), respectively, and relative humidity was 40–50%. The photoperiod was 14 h at a photon fluence rate of 300 ± 20 μmol m^-2^ s^-1^ (400–700 nm) at plant height, supplied by Philips GreenPower LED (deep red/white 120) production modules. The temperature inside the cabinets was controlled by adjusting the cabinet wall temperature. The air exchange was 40 l min^-1^ and the air inside the cabinets was stirred continuously by a ventilator. Pressurized H_2_S and SO_2_ diluted with N_2_ (1 ml l^-1^) was injected into the incoming air stream and their concentration in the cabinet was adjusted to the desired level using electronic mass flow controllers (ASM, Bilthoven, The Netherlands). The sulfur gas level in the cabinets was monitored by an SO_2_ analyzer (model 9850) equipped with a H_2_S converter (model 8770; Monitor Labs, Measurement Controls Corporation, Englewood, CO, USA). Plants were harvested 3 h after the onset of the light period and the roots were rinsed in ice-cold demineralized water (for 3 × 20 s). Roots were separated from the shoots, weighed, and for glucosinolate, myrosinase activity and RNA isolation, plant material was frozen immediately in liquid N_2_ and stored at -80°C. For analysis of dry matter, sulfate, and total sulfur, plant tissue was dried at 80°C for 24 h.

### TOTAL SULFUR AND SULFATE CONTENT

The total sulfur content was analyzed using a modification of the method as described by Jones (1995). Dried shoots and roots were pulverized in a Retsch Mixer-Mill (Retsch type MM2; Haan, Germany) and 50–150 mg of the samples was weighed into porcelain ashing trays. A 50% Mg(NO_3_)_2_ ⋅ 6H_2_O (w/v) solution was added until saturation of the material, and was dried overnight in an oven at 100°C. Subsequently, the samples were ashed in an oven at 650°C for 12 h. The residues were dissolved in 5 or 10 ml of 20% aqua regia (50 ml conc. HNO_3_ and 150 ml conc. HCl in 1 l demineralized water) and quantitatively transferred to a volumetric flask and made up to 50 or 100 ml with demineralized water. One SulphaVer® 4 Reagent Powder Pillow (HACH, Permachem® reagents, Loveland, USA) containing BaCl_2_ was added to 10 or 25 ml of extract, and the turbidity was measured with a spectrophotometer (HACH DR/400V, Loveland, CO, USA) at 450 nm. For measurement of the sulfate content, pulverized dried plant material was incubated for 3–4 h in demineralized water (10 mg/ml) at 50°C (Tausz et al., 1996; Yang et al., 2006a,b) and centrifuged at 30,000 *g* for 15 min. Anions were separated by HPLC on an Agilent IonoSpher 5A anion exchange column (250 × 4.6 mm; Agilent Technologies, Amstelveen, The Netherlands) and sulfate content was determined refractometrically according to Maas et al. (1986). The HPLC system consisted of a Knauer HPLC pump model 100 and a Knauer differential refractometer model 98.00 (Knauer, Berlin, Germany). The mobile phase contained 25 mM potassium biphthalate (pH 4.3) with 0.02% NaN_3_ (w/v). There were no significant differences in sulfate content determined in dried and fresh plant material of *B. juncea* and *B. rapa*. This indicated that there was no increase in sulfate content in dried plant material caused by the degradation of glucosinolates. The organic sulfur content was calculated by subtracting the sulfate content from the total sulfur content determined in the same tissue sample.

### GLUCOSINOLATE CONTENT

The glucosinolates were extracted and determined according to a modified method of Heaney and Fenwick (1980) and O’Callaghan et al. (2000). Frozen plant material was freeze-dried in a LyoLAB 3000 freeze drier (Heto-Holten A/S, Allerød, Denmark) for 3 days. Freeze-dried plant samples were pulverized in a Retsch Mixer-Mill (Retsch type MM2; Haan, Germany). The glucosinolates were extracted in boiling 90% methanol (50 mg in 3 ml) for 2 min. The extract was centrifuged for 2 min at 2,500 *g* and the residue was re-extracted twice with 3 ml boiling 70% methanol. Total glucosinolate content was determined based on its reaction with sodium tetrachloropalladate II (Na_2_PdCl_4_; Gupta et al., 2012; Ishida et al., 2012). The reaction mixture containing 60 μl extract and 1800 μl 2 mM Na_2_PdCl_4_ was incubated at 20°C for 30 min and the absorbance of the developed color measured colorimetrically at 450 nm (Thies, 1982). Sinigrin (Sigma–Aldrich, S1647) was used as an internal standard for all samples (13 μmol per extracts) and data were corrected for recovery rate (always higher than 80%).

### MYROSINASE ACTIVITY

The myrosinase activity was determined by the photometric quantification of released glucose with sinigrin (2-propenyl glucosinolate, S1647, Sigma–Aldrich) as substrate, as described by Travers-Martin et al. (2008). Frozen plant material was homogenized in 200 mM Tris-HCl, 10 mM EDTA, pH 7.0 (1 g fresh weight per 5 ml) at 0°C with an Ultra Turrax (T25, IKA Werke, Staufen, Germany) and filtered through one layer of Miracloth. The filtered extract was centrifuged at 16,000 *g* at 4°C for 15 min. The reaction mixture contained a final volume one ml, 150 μl supernatant and 1 mM sinigrin and was incubated at 25°C for 30 min. The reaction was stopped by incubating the reaction mixtures at 100°C for 10 min. Subsequently the reaction mixtures were centrifuged at 10,000 *g* at room temperature for 10 min and the content of glucose in the supernatant was determined by using an enzymatic glucose assay kit (Sigma–Aldrich). The soluble protein content was determined by the method of Bradford (1976) using bovine serum albumin as a standard.

### RNA EXTRACTION AND REAL-TIME QUANTITATIVE PCR OF MYROSINASE

Total RNA was isolated by a modified hot phenol method (Verwoerd et al., 1989). Frozen ground plant material was extracted in hot (80°C) phenol/extraction buffer (1:1, v/v), 1 g ml^-1^. The extraction buffer contained 0.1 M Tris-HCl, 0.1 M LiCl, 1% SDS (w/v), 10 mM EDTA, pH 8.0). After mixing, 0.5 ml of chloroform–isoamyl alcohol (24:1, v/v) was added. After centrifugation (13,400 *g*) for 5 min at 4°C, the aqueous phases were transferred to new tube. After adding an equal volume of chloroform and isoamyl alcohol, the total RNA was precipitated by 4 M LiCl overnight at 4°C. Total RNA was collected and washed with 70% ethanol. Possible genomic DNA contamination was removed with a DNase treatment step (Promega, USA). Phenol–chloroform–isoamyl alcohol and chloroform–isoamyl alcohol were used for further purification and total RNA was precipitated by ethanol and dissolved in diethylpyrocarbonate-treated water. The quantity and quality of RNA was checked using ThermoNanoDrop 2000 and RNA in each was adjusted to the same concentration. The integrity of RNA was checked by electrophoresis by loading 1 μg RNA on a 1% TAE-agarose gel.

DNA-free intact RNA (1 μg) was reverse transcribed into cDNA with oligo-dT primers using a first strand cDNA synthesis kit (Promega, USA) according to the manufacture-supplied instructions. Subsequently, the cDNA was used as a template in real-time PCR experiments with gene-specific primers. To design primers, the full length complementary DNA of the *Arabidopsis* genes, actin (reference gene) and myrosinase (TGG1 and TGG2), which are mostly expressed in the shoot (Andersson et al., 2009), were used to query homologous *B. juncea* and *B. rapa* sequences. Expressed sequence tags (ESTs) were obtained from the publically available platform at NCBI. The primer sequences used for myrosinase and actin were (F 5′-CCGGTCGATGTTCTCCTAT-3′, R 5′-GAAGAATTTCCACCGTAACAC-3′) and (F 5′-AGCAGCATGAAGATCAAGGT-3′, R 5′-GCTGAGGGATGCAAGGATAG-3′), respectively. RT-PCR was performed on Applied Bio Systems’ 7300 real-time PCR system using the SYBR Green master mix kit (Thermo Scientific) based on manufacturer’s instructions. The transcript level of the target gene and actin was measured using the comparative Ct method. Analysis of qPCR data was performed using three independent RNA preparations from separate plant shoots.

### STATISTICAL ANALYSIS

Data from different experimental sets ware analyzed for statistical significance using an unpaired two-tailed Student’s *t*-test (*P* < 0.01).

## RESULTS

### IMPACT OF ATMOSPHERIC AND PEDOSPHERIC SULFUR NUTRITION ON GROWTH AND SULFUR CONTENT

**FIGURE 1:**
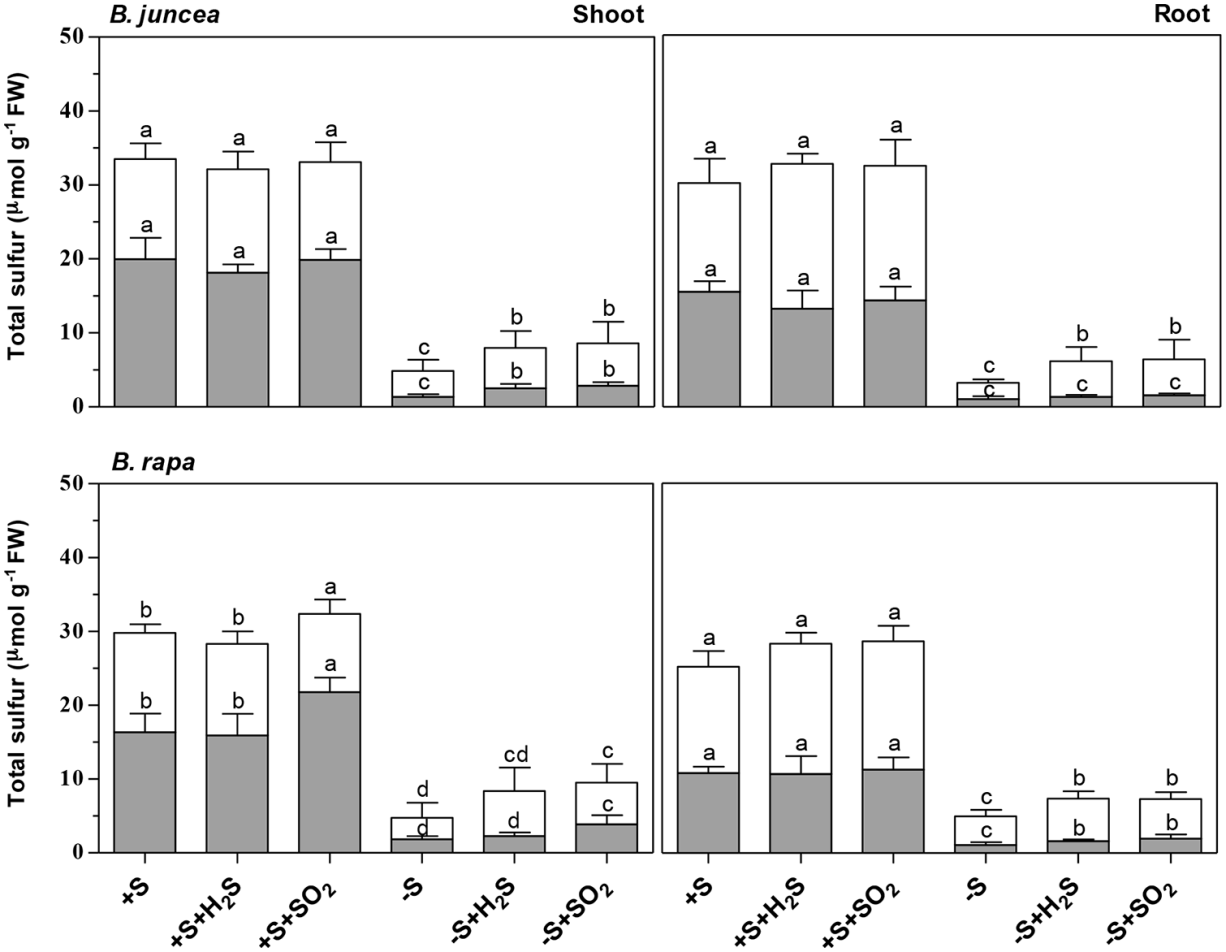
**Impact of H_2_S, SO_2_ and sulfate deprivation on total sulfur, sulfate and organic sulfur content of shoots and roots of *B. juncea* and *B. rapa*.** For experimental details, see legends of **Table 1**. The sulfate and organic sulfur fraction is presented in gray and white, respectively. Data represent the mean of two experiments with three measurements with six to nine plants in each (±SD). Different letters indicate significant differences between treatments (*P* < 0.01, Student’s *t*-test).

Exposure of *B. juncea* and *B. rapa* to 0.25 μl l^-1^ H_2_S and 0.25 μl l^-1^ SO_2_ for 7 days did not significantly affect plant biomass production, shoot to root ratio, and dry matter content (DMC) of shoots and roots at sulfate-sufficient conditions (**Table 1**). In addition, H_2_S exposure did not affect the total sulfur, sulfate, and organic sulfur content of the shoots and roots of both species (**Figure 1**). Moreover SO_2_ exposure also did not affect the sulfur, sulfate, and organic sulfur content of both shoots and roots of *B. juncea*. In *B. rapa*, however, SO_2_ exposure resulted in a slight but significant increase in total sulfur content of the shoot, which could be attributed to an increase in the sulfate content, whereas the sulfur content of the root remained unaffected. Furthermore the organic sulfur content was hardly affected by the exposure of *B. rapa* to SO_2_ in sulfate-sufficient conditions in both shoots and roots (**Figure 1**).

**Table 1:** Impact of H_2_S, SO_2_ and sulfate deprivation on biomass production and dry matter content (DMC) of shoots and roots of *B. juncea* and *B. rapa*.

	+S	+S + H_2_S	+S + SO_2_	-S	-S + H_2_S	-S + SO_2_
***B. juncea***
Shoot biomass production	0.71 ± 0.18a	0.75 ± 0.27a	0.65 ± 0.19a	0.34 ± 0.08b	0.66 ± 0.10a	0.71 ± 0.17a
Root biomass production	0.14 ± 0.05c	0.13 ± 0.04c	0.13 ± 0.05c	0.16 ± 0.06bc	0.20 ± 0.05ab	0.23 ± 0.09a
Shoot DMC	10.4 ± 1.1a	9.8 ± 0.8a	10.4 ± 1.1a	11.3 ± 1.1a	9.7 ± 0.6a	10.0 ± 0.5a
Root DMC	8.9 ± 0.7a	7.9 ± 1.7ab	8.1 ± 1.3a	6.1 ± 1.0b	6.8 ± 0.7b	5.8 ± 0.8b
Shoot/root ratio	5.3 ± 0.7a	5.7 ± 1.2a	5.2 ± 0.9a	2.2 ± 0.5c	3.9 ± 0.6b	3.0 ± 0.6bc
***B. rapa***
Shoot biomass production	1.14 ± 0.28a	1.07 ± 0.27a	0.90 ± 0.26ab	0.37 ± 0.17c	0.74 ± 0.30b	0.78 ± 0.22b
Root biomass production	0.19 ± 0.06ab	0.16 ± 0.05b	0.15 ± 0.04b	0.13 ± 0.06c	0.19 ± 0.07ab	0.23 ± 0.08a
Shoot DMC	9.5 ± 0.5b	9.3 ± 0.6b	9.8 ± 0.4b	11.7 ± 1.2a	9.6 ± 0.8b	9.8 ± 0.7ab
Root DMC	7.2 ± 1.4a	7.8 ± 1.3a	7.3 ± 0.8a	8.2 ± 2.3a	7.5 ± 1.1a	7.2 ± 0.6a
Shoot/root ratio	6.0 ± 1.0a	7.0 ± 1.2a	6.2 ± 0.5a	2.9 ± 0.8c	4.0 ± 0.8bc	3.5 ± 0.6bc

*Ten day-old seedlings were grown on a 25% Hoagland solution containing 0.5 mM sulfate for 3 days and subsequently transferred to fresh 25% Hoagland solution at 0 (–S) or 0.5 mM sulfate (+S) and exposed to 0.25 μl l ^*–1*^ H_2_S or SO_2_ for 7 days. The initial fresh weight of shoots and roots in B. juncea were 0.042 ± 0.008 g and 0.022 ± 0.003 g, respectively. The initial fresh weight of shoots and roots in B. rapa were 0.066 ± 0.025 g and 0.021 ± 0.003 g, respectively. Data on biomass production (g FW), shoot/root ratio and DMC (%) represent the mean of four independent experiments with three measurements with six to nine plants in each (±SD). Different letters indicate significant differences between treatments (P < 0.01, Student’s t-test).*

Sulfate deprivation resulted in a decreased biomass production of *B. juncea* and *B. rapa* (**Table 1**). However, shoot growth was more affected than root growth, resulting in a decrease on the shoot to root ratio upon sulfate deprivation. A 7-day sulfate deprivation resulted in 53 and 68% decreases of shoot biomass production of *B. juncea* and *B. rapa*, respectively. The root biomass production of *B. juncea* was not affected and that of *B. rapa* was reduced by 32%. The DMC of the shoot of *B. juncea* was not affected by sulfate deprivation, but that of the root was decreased (**Table 1**). However, the DMC of the shoot of *B. rapa* was significantly increased, whereas that of the root was hardly affected (**Table 1**). Sulfate deprivation resulted in strongly decreased total sulfur, sulfate, and organic sulfur contents of shoots and roots of both species (**Figure 1**). In particular, the proportion of sulfate was diminished and utilized for the synthesis for organic sulfur compounds (**Figure 1**).

Exposure of plants to 0.25 μl l^-1^ H_2_S and SO_2_ alleviated either fully (*B. juncea*) or largely (*B. rapa*) the decrease in shoot biomass production upon sulfate-deprivation, demonstrating that the foliarly absorbed sulfur gases replaced sulfate taken up by the root as a sulfur source for growth. However, the root biomass production of H_2_S and SO_2_ exposed sulfate-deprived plants was even higher than that of plants at sulfate-sufficient conditions (**Table 1**). As a consequence, the shoot to root ratio was lower for sulfate-deprived H_2_S and SO_2_ exposed plants than that of sulfate-sufficient plants. The shoot DMC of *B. juncea* for sulfate-deprived plants was hardly affected by H_2_S and SO_2_ exposure and was quite similar to that of sulfate-sufficient plants (**Table 1**). The DMC of the root sulfate-deprived H_2_S and SO_2_ exposed plants remained lower than that of sulfate-sufficient plants. The DMC of the shoots and roots of sulfate-deprived *B. rapa* was only slightly affected by H_2_S and SO_2_ exposure and was quite similar to that of sulfate-sufficient plants (**Table 1**).

Both H_2_S and SO_2_ exposure of sulfate-deprived *B. juncea* and *B. rapa* resulted in an increase in total sulfur and organic sulfur content of both shoots and roots (**Figure 1**) however the overall total sulfur content was almost threefold and 2.5-fold lower than that observed for sulfate-sufficient *B. juncea* and *B. rapa*, respectively. The low total sulfur content could only in part be ascribed to a low apparent sulfate content upon sulfate-deprivation.

### IMPACT OF ATMOSPHERIC AND PEDOSPHERIC SULFUR NUTRITION ON GLUCOSINOLATE CONTENT

**FIGURE 2:**
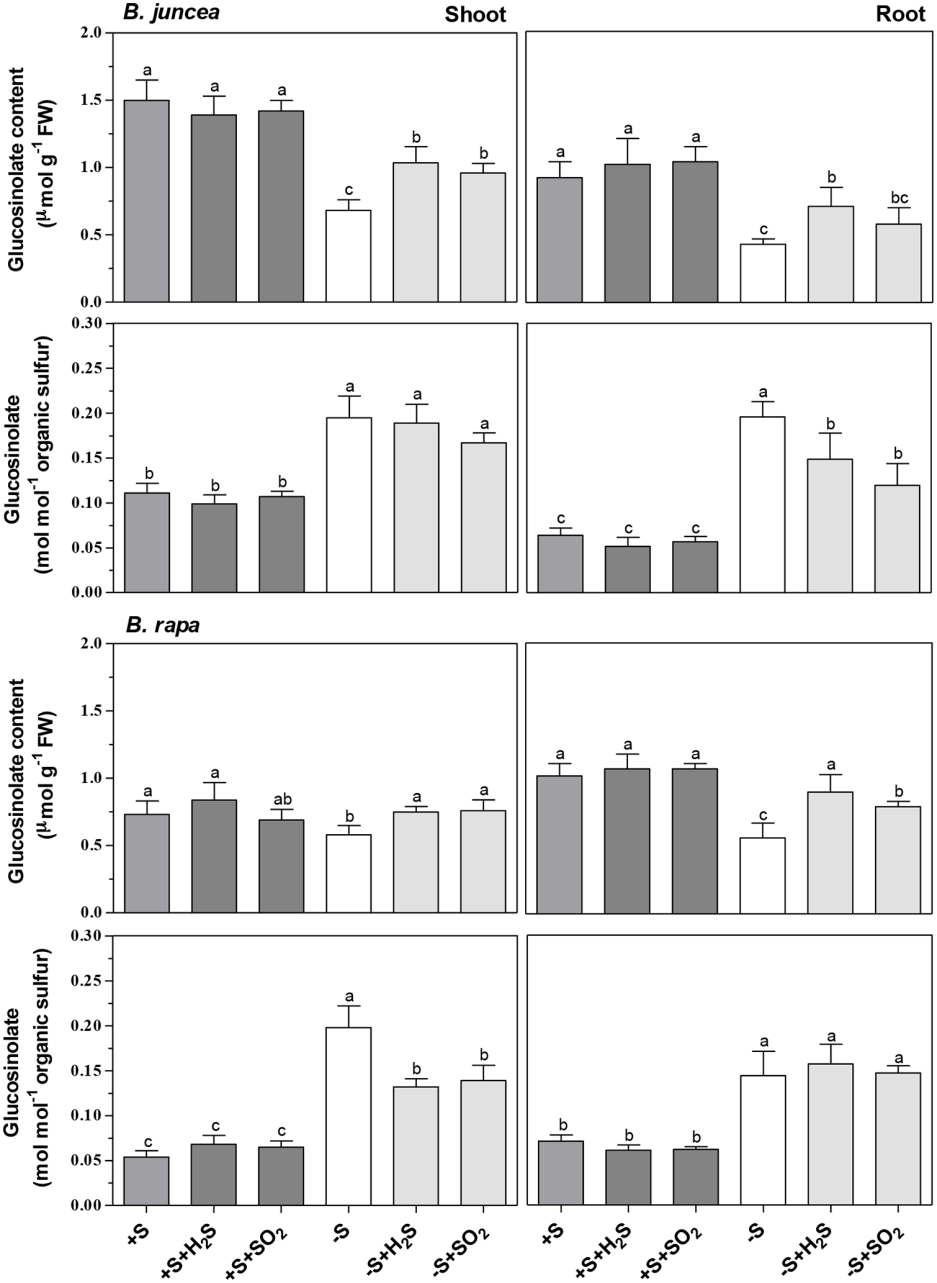
**Impact of H_2_S, SO_2_ and sulfate deprivation on glucosinolate content of shoots and roots of *B. juncea* and *B. rapa*.** For experimental details, see legends of **Table 1**. Data represent the mean of two experiments with three measurements with nine plants in each (±SD). Different letters indicate significant differences between treatments (*P* < 0.01, Student’s *t*-test).

There were considerable differences in the content of glucosinolates of shoots of *B. juncea* and *B. rapa* seedlings. The glucosinolate content of the shoot of *B. juncea* was twofold higher than that in the shoot of *B. rapa*, expressed either on a fresh weight basis and or relative to organic sulfur (**Figure 2**). However, the glucosinolate content of the roots of both *Brassica* species was quite similar. Neither H_2_S and SO_2_ exposure affected the glucosinolate content of shoots and roots of *B. juncea* and *B. rapa*, expressed either on a fresh weight or organic sulfur basis (**Figure 2**).

A 7-day sulfate deprivation resulted in 50% decrease in the glucosinolate content of both shoots and roots of *B. juncea* and a 20 and 45% decrease in content of the shoots and roots of *B. rapa*, respectively (**Figure 2**). H_2_S and SO_2_ exposure of sulfate-deprived plants alleviated almost fully the decrease in glucosinolate contents of shoot and roots of *B. rapa*, and their contents were quite similar (except that of the roots of sulfate-deprived SO_2_-exposed plants) to that of sulfate-sufficient plants (**Figure 2**). H_2_S and SO_2_ exposure of sulfate-deprived *B. juncea* also resulted in a higher glucosinolate content of both shoots and roots. However, their contents were significantly lower than that observed in sulfate-sufficient plants (**Figure 2**). On an organic sulfur basis, however, the glucosinolate content of shoots and roots of sulfate-deprived of *B. juncea* and *B. rapa*, both in absence and presence of H_2_S or SO_2_, was always higher than that observed in sulfate-sufficient plants (**Figure 2**). This indicated that proportionally, the content of other organic sulfur compounds (e.g., proteins), was more affected by sulfate deprivation than that of the glucosinolates, even in presence of foliarly absorbed H_2_S or SO_2_ as alternative sulfur sources for growth.

### IMPACT OF ATMOSPHERIC AND PEDOSPHERIC SULFUR NUTRITION ON THE ACTIVITY AND EXPRESSION OF MYROSINASE

**FIGURE 3:**
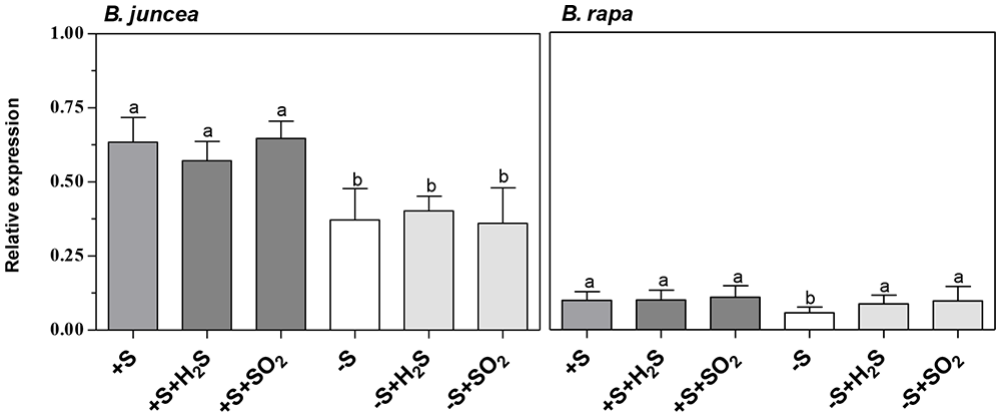
**Impact of H_2_S, SO_2_ and sulfate deprivation on transcript levels of myrosinase in shoots of *B. juncea* and *B. rapa.*** For experimental details, see legends of **Table 1**. Relative gene expression of myrosinase was determined by qRT-PCR and the mRNA levels were compared to actin. Data on relative expression in each treatment represent the mean of three measurements with three shoots in each (±SD). Different letters indicate significant differences between treatments (*P* < 0.01, Student’s *t*-test).

There were considerable differences in the activity of myrosinase in shoots and roots of *B. juncea* and *B. rapa* seedlings (**Table 2**). Both shoots and roots of *B. juncea* were characterized by a high myrosinase activity. *B. rapa,* however, was characterized by very low and hardly detectible myrosinase activity in the shoot and high activity in the root, the latter being quite similar to that observed in *B. juncea*. If glucoberin was used as substrate instead of sinigrin, comparable myrosinase activities were observed (data not presented). This demonstrated that the differences in activity of this enzyme in the shoots of *B. juncea* and *B. rapa* were unlikely to be explained by differences in substrate selectivity. Additionally the transcript level of myrosinase was substantially lower in shoots of *B. rapa* than that of *B. juncea* (**Figure 3**). In sulfate-sufficient conditions, H_2_S and SO_2_ exposure did not affect the activities of myrosinase enzyme of shoots and roots of *B. juncea* and *B. rapa* (**Table 2**) or the expression of the gene in the shoots (**Figure 3**).

**Table 2:** Impact of H_2_S, SO_2_ and sulfate deprivation on myrosinase activity and water-soluble protein content of shoots and roots of *B. juncea* and *B. rapa*. For experimental details see legends of **Table 1**.

	+S	+S+ H_2_S	+S+ SO_2_	-S	-S+ H_2_S	-S+ SO_2_
***B. juncea***
**Shoot**
Myrosinase activity	0.31 ± 0.03ab	0.28 ± 0.01b	0.34 ± 0.03ab	0.37 ± 0.02a	0.36 ± 0.02a	0.38 ± 0.02a
Soluble proteins content	13.5 ± 1.7a	12.9 ± 1.8a	13.7 ± 1.2a	9.5 ± 1.0b	12.1 ± 1.3a	12.5 ± 1.6a
**Root**
Myrosinase activity	0.34 ± 0.07a	0.27 ± 0.04a	0.26 ± 0.06a	0.10 ± 0.01b	0.07 ± 0.01c	0.11 ± 0.03bc
Soluble proteins content	4.1 ± 0.5a	4.2 ± 1.0a	3.7 ± 0.9a	1.1 ± 0.1c	2.3 ± 0.4b	2.7 ± 0.48ab
***B. rapa***
**Shoot**
Myrosinase activity	<0.01a	<.0.01a	<0.01a	<0.01a	<0.01a	<0.01a
Soluble proteins content	11.4 ± 1.3a	12.6 ± 2.1a	11.8 ± 1.0a	8.6 ± 0.5b	10.5 ± 0.6a	11.6 ± 1.4a
**Root**
Myrosinase activity	0.42 ± 0.03a	0.48 ± 0.07a	0.40 ± 0.03a	0.14 ± 0.01b	0.12 ± 0.02b	0.13 ± 0.02b
Soluble proteins content	4.2 ± 0.3a	4.9 ± 1.1ab	4.3 ± 0.7ac	1.2 ± 0.1d	2.5 ± 0.2bc	3.0 ± 0.1b

*For experimental details see legends of **Table 1**. Data on myrosinase activity (nmol mg^-1^ fresh weight min^-1^) and soluble protein content (mg g^-1^ fresh weight)represent the mean of three measurements with three plants in each (±SD). Different letters indicate significant differences between treatments (*P* < 0.01, Student’s *t*-test).*

A 7-day sulfate deprivation resulted in a decrease in myrosinase activity in roots of *B. juncea* and *B. rapa* (expressed on a fresh weight basis), while activity in the shoot of *B. juncea* was not affected (**Table 2**). However, the transcript levels of myrosinase in shoots of both *B. juncea* and *B. rapa* were decreased upon sulfate deprivation (**Figure 3**). Sulfate deprivation resulted in a decrease in soluble protein content of shoots and roots of both species (**Table 2**). If the myrosinase activity in the roots of both species was expressed on a protein basis, then specific activity remained unaffected and was around 90 nmol mg^-1^ protein min^-1^, whereas in the shoot of *B. juncea* its activity increased from 23 in sulfate-sufficient to 39 nmol mg^-1^ protein min^-1^ in sulfate-deprived plants. Exposure of sulfate-deprived plants to H_2_S or SO_2_ resulted in an increase in soluble protein content, although it hardly affected the myrosinase activity in shoots and roots of *B. juncea* and *B. rapa* (expressed on a fresh weight basis; **Table 2**). H_2_S or SO_2_ exposure of sulfate-deprived plants did not affect the myrosinase transcript level in the shoot of *B. juncea*, whereas it was increased in the shoot of *B. rapa* (**Figure 3**).

## DISCUSSION

Brassicaceae are fast growing species characterized by a relatively high sulfur requirement (Westerman et al., 2001a; Castro et al., 2003; Yang et al., 2006a,b). Under the experimental conditions used here, *B. juncea* and *B. rapa* seedlings had a relative growth rate of 36.9 and 38.6% day^-1^ and a plant sulfur content of 33.4 and 29.4 μmol g^-1^ fresh weight (340 and 272 μmol g^-1^ dry weight), respectively (data derived from **Table 1**; **Figure 1**). Seedlings of Brassicaceae are often characterized by a relatively high ratio of sulfate to organic sulfur. For instance, in *B. oleracea* the sulfate content may account for more than 80% of the total sulfur in the shoot (Westerman et al., 2001b; Castro et al., 2003; Yang et al., 2006a,b). The sulfate content accounted for 60 and 50% of total sulfur in the shoots and roots of *B. juncea*, and for 55 and 43% in shoots and roots of *B. rapa*, respectively. *Brassica* species are also characterized by a relatively high content of secondary sulfur compounds, viz. glucosinolates, which strongly varies between species, and may in seedlings account for 10 to 23% of the organic sulfur fraction (Castro et al., 2004). The glucosinolates content of the shoot of *B. juncea* was twofold higher than that in the shoot of *B. rapa* and accounted for up to 30% of the organic sulfur fraction (on the basis of 3 S groups per molecule), presuming that the aliphatic glucosinolates are the major secondary sulfur compounds present in *Brassica* (Kushad et al., 1999; Van Dam et al., 2003; Cartea et al., 2008). In the root, content appeared to be quite similar in both species, where it accounted for 15% of the organic sulfur fraction.

Atmospheric sulfur gases, viz. SO_2_ and H_2_S, may be taken up by the plant shoot and used as a sulfur source for growth (De Kok et al., 2007). The foliar uptake of SO_2_ is determined by its chemical/physical properties, viz. rapid dissociation in the water of the mesophyll apoplast, which is beyond regulatory control (De Kok et al., 2007). The foliar uptake of H_2_S, however, is largely determined by the rate of metabolism in the shoot (De Kok et al., 2007). Exposure of *B. juncea* and *B. rapa* to 0.25 μl l^-1^ SO_2_ and H_2_S hardly affected the total sulfur content of both species. There was only a slight increase in the shoot total sulfur content of SO_2_-exposed *B. rapa*, which could be attributed to an enhanced sulfate content. However, it was evident that at this atmospheric sulfur concentration of SO_2_ and H_2_S, plants were able to take up sufficient sulfur by the shoot to fully cover their organic sulfur requirement for plant growth. Since, the decrease in biomass production upon sulfate-deprivation was completely alleviated by SO_2_ and H_2_S exposure. Similarly to previous observations, sulfate-deprived plants invested relatively more biomass in their roots than those grown under sulfate-sufficient conditions, even upon SO_2_ and H_2_S exposure (Buchner et al., 2004; Yang et al., 2006a; Koralewska et al., 2008; Shahbaz et al., 2014).

In greenhouse and field experiments involving soil, hydroponic, and tissue culture media, sulfur fertilization generally resulted in an increased glucosinolate content of *Brassica* (Falk et al., 2007). However, SO_2_ and H_2_S exposure of *B. juncea* and *B. rapa* seedlings did not affect the glucosinolate content of the shoots and roots, either expressed on fresh weight or organic sulfur basis. This demonstrated that these sulfur compounds did not form a sink for excessive supplied atmospheric sulfur. SO_2_ and H_2_S exposure of *Brassica* generally resulted in enhanced levels of water-soluble, non-protein thiol compounds in the shoot (De Kok and Tausz, 2001; Westerman et al., 2001a; Buchner et al., 2004; Yang et al., 2006a; Koralewska et al., 2008; Shahbaz et al., 2014), which could be ascribed to an accumulation of cysteine and glutathione (De Kok and Tausz, 2001; De Kok et al., 2007). Apparently, an enhanced availability of these thiol compounds, which also function as reduced sulfur donors in the synthesis of glucosinolates (Schnug, 1990, 1993; Halkier and Gershenzon, 2006; Falk et al., 2007) did not affect the rate of synthesis of these secondary sulfur compounds in the shoot. *Allium* species (e.g., onion, garlic, leek) also contain secondary sulfur compounds viz. γ-glutamyl peptides and allins, which are synthesized from cysteine, via γ-glutamylcysteine or glutathione. In contrast to observations with glucosinolates, in *Brassica* the content of these secondary sulfur compounds, their precursors and/or degradation products were strongly enhanced in shoots of H_2_S-exposed *Allium* (Durenkamp and De Kok, 2002, 2004; Durenkamp et al., 2005). SO_2_ exposure, however, hardly affected the levels of these secondary sulfur compounds in *Allium* (Durenkamp et al., 2005).

Exposure of *Brassica* to H_2_S resulted in a strong decrease in expression, in protein level and in enzyme activity of APS reductase in the shoot (Westerman et al., 2000a,b, 2001a,b; Durenkamp et al., 2007; Koralewska et al., 2008; Shahbaz et al., 2014). Evidently, a down-regulation of APS reductase, the key enzyme controlling the flux through the sulfate reduction pathway, did not affect the synthesis of glucosinolates via the channeling of the APS through the APS kinase/sulfotransferase pathway. The latter is essential for the synthesis of the sulfated moiety of the glucosinolates (Kopriva et al., 2012). This demonstrated that the synthesis of glucosinolates in the shoot of *Brassica* seedlings was under strict regulatory control and was not affected by an excess supply of foliarly absorbed sulfur, irrespective of the differences in content of these secondary sulfur compounds between *B. juncea* and *B. rapa*.

Sulfate deprivation resulted in a strong decrease in the total sulfur content of shoots and roots of *B. juncea* and *B. rapa*, which was for the greater part due to a decrease in sulfate content. It has been observed that in *Arabidopsis* sulfur deficiency resulted in a repression of the glucosinolate biosynthesis genes (Hirai et al., 2005). There was also a strong decrease in the glucosinolate content of both shoots and roots of *B. juncea* and *B. rapa* upon sulfate deprivation. However, the decrease in glucosinolate content was lower than that of the other organic sulfur compounds, resulting in an increase in its content expressed on organic sulfur basis. Sulfur in proteins generally accounts for more than 80% of the organic sulfur content (Stulen and De Kok, 1993). Apparently, sulfate deprivation had a higher impact on the proportion of sulfur in proteins in *Brassica* than the other organic sulfur compounds viz. glucosinolates. It has been observed that in shoots, sulfate deprivation resulted in a degradation of Rubisco, which proportion may account for 25–60% of the soluble proteins in photosynthetic tissue (Ferreira and Teixeira, 1992; Gilbert et al., 1997). The proportion of sulfur in the glucosinolates in both shoots and roots of *B. juncea* and *B. rapa* in sulfate-deprived plants exceeded 50% of the total organic sulfur fraction (on the basis of 3 S groups per molecule). These results indicated that glucosinolates cannot be considered as sulfur storage compounds and they were not utilized in the re-distribution of sulfur in *B. juncea* and *B. rapa* seedlings upon sulfate deprivation.

SO_2_ and H_2_S exposure resulted in an increase in total sulfur content of shoots and roots of the two *Brassica* species upon sulfate deprivation, which was mainly due to an increase in the organic sulfur fraction. Likewise, there was an increase in glucosinolate content, though the levels were always lower than that of sulfate-sufficient plants. However, on an organic sulfur basis, the glucosinolate content in both shoots and roots was substantially higher than that observed in sulfate-sufficient plants upon SO_2_ and H_2_S exposure. Apparently in sulfate-deprived conditions, a relatively greater proportion of atmospheric sulfur taken up by the shoot was used for the synthesis of glucosinolates than that of other organic sulfur compounds, e.g., proteins. Roots of sulfate-deprived plants depended on sulfur transported form shoot to root upon exposure to SO_2_ and H_2_S, although in which form the sulfur was transported from shoot to root under these conditions remains unknown. Organic sulfur may be transported in the phloem from source to sink, e.g., from shoot to root in different forms, viz. glutathione, *S*-methylmethionine or as glucosinolates (Brunold and Rennenberg, 1997; Bourgis et al., 1999; Grubb and Abel, 2006; Rennenberg and Herschbach, 2014).

Glucosinolates may be involved in plant defense against pathogens and herbivory (Bones et al., 1994; Brader et al., 2006; Grubb and Abel, 2006; Bednarek et al., 2011; Frerigmann et al., 2012; Schiestl, 2014) and is dependent upon breakdown activated by tissue damage and catalyzed by myrosinase (Bones et al., 1994; Grubb and Abel, 2006; Clay et al., 2009; Kissen and Bones, 2009; Kissen et al., 2009; Ahuja et al., 2010). Moreover, it has been suggested that myrosinase might have significance in the redistribution of sulfur in plants under sulfur-deprived conditions (Schnug, 1990; Hirai et al., 2004, 2005; Bloem et al., 2007; Falk et al., 2007).

There was a direct relation between the content of glucosinolates and the transcript level and activity of myrosinase in the shoot of *B. juncea* and *B. rapa*. Both low glucosinolate content and a low transcript level of myrosinase and activity of this enzyme characterized the shoot of the latter species. Even though myrosinase activity was decreased in both roots of sulfate-deprived *B. juncea* and *B. rapa*, SO_2_ and H_2_S exposure hardly affected the activity in shoots and roots under either sulfate-sufficient or sulfate-deprived conditions, despite an increase in glucosinolate content upon exposure in sulfate-deprived plants. There was apparently no direct co-regulation between the content of glucosinolates and the activity of myrosinase. From the current observation that the sulfur of glucosinolates was hardly re-distributed upon sulfate deprivation, the significance of myrosinase in the *in situ* turnover of these secondary sulfur compounds needs to be questioned.

## AUTHOR CONTRIBUTIONS

Tahereh Aghajanzadeh, Malcolm J. Hawkesford, and Luit J. De Kok designed the research. Tahereh Aghajanzadeh carried out the experiments and analyzed the data. All authors contributed to writing the manuscript. Luit J. De Kok and Malcolm J. Hawkesford supervised the project.

## Conflict of Interest Statement

The authors declare that the research was conducted in the absence of any commercial or financial relationships that could be construed as a potential conflict of interest.

## References

[B1] Ahuja I. Rohloff J. Bones A. M. (2010). Defence Mechanisms of Brassicaceae: implications for plant-insect interactions and potential for integrated pest management. *Agron. Sustain. Dev.* 30 311–348 10.1051/Agro/2009025

[B2] Andersson D. Chakrabarty R. Bejai S. Zhang J. Rask L. Meijer J. (2009). Myrosinases from root and leaves of *Arabidopsis thaliana* have different catalytic properties. *Phytochemistry* 70 1345–1354 10.1016/j.phytochem.2009.07.036 19703694

[B3] Antonious G. Bomford M. Vincelli P. (2009). Screening *Brassica* species for glucosinolate content. *J. Environ. Sci. Health B* 44 311–316 10.1080/03601230902728476 19280485

[B4] Bednarek P. Piślewska-Bednarek M. van Themaat E. V. L. Maddula R. K. Svatoš A. Schulze-Lefert P. (2011). Conservation and clade-specific diversification of pathogen-inducible tryptophan and indole glucosinolate metabolism in *Arabidopsis thaliana* relatives. *New Phytol.* 192 713–726 10.1111/j.1469-8137.2011.03824.x 21793828

[B5] Bloem E. Haneklaus S. Schnug E. (2007). Changes in the sulphur and glucosinolate content of developing pods and seeds of oilseed rape(*Brassica napus* L.) in relation to different cultivars. *Landbauforsch. Völk.* 57 297–306.

[B6] Bones A. M. Rossiter J. T. (1996). The myrosinase-glucosinolate system, its organisation and biochemistry. *Physiol. plant.* 96 194–208 10.1111/j.1399-3054.1996.tb00497.x

[B7] Bones A. M. Rossiter J. T. (2006). The enzymic and chemically induced decomposition of glucosinolates. *Phytochemistry* 67 1053–1067 10.1016/j.phytochem.2006.02.024 16624350

[B8] Bones A. Visvalingam S. Thangstad O. (1994). Sulphate can induce differential expression of thioglucoside glucohydrolases (myrosinases). *Planta* 193 558–566 10.1007/BF02411562

[B9] Bourgis F. Roje S. Nuccio M. L. Fisher D. B. Tarczynski M. C. Li C. (1999). S-methylmethionine plays a major role in phloem sulfur transport and is synthesized by a novel type of methyltransferase. *Plant Cell* 11 1485–1497 10.1105/tpc.11.8.1485 10449582 PMC144290

[B10] Brader G. Mikkelsen M. D. Halkier B. A. Palva E. T. (2006). Altering glucosinolate profiles modulates disease resistance in plants. *Plant J.* 46 758–767 10.1111/j.1365-313X.2006.02743.x 16709192

[B11] Bradford M. M. (1976). A rapid and sensitive method for the quantitation of microgram quantities of protein utilizing the principle of protein-dye binding. *Anal. Biochem.* 72 248–254 10.1016/0003-2697(76)90527-3 942051

[B12] Brunold C. Rennenberg H. (1997). “Regulation of sulfur metabolism in plants: first molecular approaches,” in *Progress in Botany,* eds Behnke H. D. Lüttge U. Karl Esser H. C. M. Kadereit J. W. Runge M. (Berlin: Springer) 58 164–186.

[B13] Buchner P. Stuiver C. E. E. Westerman S. Wirtz M. Hell R. Hawkesford M. J. (2004). Regulation of sulfate uptake and expression of sulfate transporter genes in *Brassica oleracea* as affected by atmospheric H_2_S and pedospheric sulfate nutrition. *Plant Physiol.* 136 3396–3408 10.1104/pp.104.046441 15377780 PMC523398

[B14] Cartea M. E. Velasco P. Obregón S. Padilla G. De Haro A. (2008). Seasonal variation in glucosinolate content in *Brassica oleracea* crops grown in northwestern Spain. *Phytochemistry* 69 403–410 10.1016/j.phytochem.2007.08.014 17889044

[B15] Castro A. Aires A. Rosa E. Bloem E. Stulen I. (2004). Distribution of glucosinolates in *Brassica oleracea* cultivars. *Phyton (B Aires)* 44 133–143.

[B16] Castro A. Stulen I. De Kok L. J. (2003). “Nitrogen and sulfur requirement of *Brassica oleracea* L. cultivars,” in *Sulfur Transport and Assimilation in Plants*, eds Davidian J. C. Grill D. De Kok L. J. Stulen I. Hawkesford M. J. Schnug E. (Leiden: Backhuys Publishers), 181–183.

[B17] Clay N. K. Adio A. M. Denoux C. Jander G. Ausubel F. M. (2009). Glucosinolate metabolites required for an *Arabidopsis* innate immune response. *Science* 323 95–101 10.1126/science.1164627 19095898 PMC2630859

[B18] De Kok L. J. Durenkamp M. Yang L. Stulen I. (2007). “Atmospheric sulfur,” in *Sulfur in Plants: An Ecological Perspective*, eds Hawkesford M. J. De Kok L. J. (Berlin: Springer), 91–106 10.1007/978-1-4020-5887-5_5

[B19] De Kok L. J. Stuiver C. E. E. Westerman S. Stulen I. (2000a). “Atmospheric H_2_S pollution – deposition and impact on sulphur metabolism in plants,” in *Environmental Stress: Indication, Mitigation and Eco-Conservation*, eds Yunus M. Singh N. De Kok L. J. (Berlin: Springer), 135–141.

[B20] De Kok L. J. Westerman S. Stuiver C. E. E. Stulen I. (2000b). “Atmospheric H_2_S as plant sulphur source: interaction with pedospheric sulfur nutrition - a case study with *Brassica oleracea* L.,” in *Sulphur Nutrition and Sulphur Assimilation in Higher Plants: Molecular, Biochemical and Physiological Aspects*, eds Brunold C. Rennenberg H. De Kok L. J. Stulen I. Davidian J.-C. (Bern: Paul Haupt Verlag), 41–55.

[B21] De Kok L. J. Stuiver C. E. E. Westerman S. Stulen I. (2002). “Elevated level of hydrogen sulphide in the plant environment: nutrient or toxin,”in *Air Pollution and Plant Biotechnology-Prospects for Phytomonitoring and Phytoremediation*, eds Omasa K. Saji H. Youssefian S. Kondo N. (Tokyo: Springer-Verlag) 201–219.

[B22] De Kok L. J. Tausz M. (2001). “The role of glutathione in plant reaction and adaptation to air pollutants,” in *Significance of Glutathione to Plant Adaptation to the Environment*, eds Grill D. Tausz M. De Kok L. J. (Dordrecht: Kluwer Academic Publishers) 185–201.

[B23] De Kok L. J. Yang L. Stuiver C. E. E. Stulen I. (2009). “Negative versus positive functional plant responses to air pollution: a study establishing cause-effect relationship of SO_2_ and H_2_S,” in *Air Quality and Ecological Impacts: Relating Sources to Effects*, ed.Legge A. K. (Amsterdam: Elsevier), 121–134.

[B24] Del Carmen Martinez-Ballesta M. Moreno D. Carvajal M. (2013). The physiological importance of glucosinolates on plant response to abiotic stress in *Brassica*. *Int. J. Mol. Sci.* 14 11607–11625 10.3390/ijms140611607 23722664 PMC3709749

[B25] Durenkamp M. De Kok L. J. (2002). The impact of atmospheric H_2_S on growth and sulfur metabolism of *Allium* cepa L. *Phyton (B Aires)* 42 55–63.

[B26] Durenkamp M. De Kok L. J. (2004). Impact of pedospsheric and atmospheric sulphur nutrition on sulphur metabolism of *Allium* cepa L. a species with a potential sink capacity for secondary sulphur compounds. *J. Exp. Bot.* 55 1821–1830 10.1093/jxb/erh187 15234992

[B27] Durenkamp M. De Kok L. J. Kopriva S. (2007). Adenosine 5’-phosphosulphate reductase is regulated differently in *Allium cepa* L. and *Brassica oleracea* L. upon exposure to H_2_S. *J. Exp. Bot.* 58 1571–1579 10.1093/jxb/erm031 17332418

[B28] Durenkamp M. Posthumus F. S. Stuiver C. E. E. De Kok L. J. (2005). “Metabolism of atmospheric sulfur gases in onion,” in *Plant Responses to Air Pollution and Global Change*, eds Omasa K. Nouchi I. De Kok L. J. (Tokyo: Springer-Verlag), 3–11.

[B29] Ernst W. (1993). “Ecological aspects of sulfur in higher plants: The impact of SO_2_ and the evolution of the biosynthesis of organic sulfur compounds on populations and ecosystems,” in *Sulfur Nutrition and Sulfur Assimilation in Higher Plants; Regulatory, Agricultural and Environmental Aspects*, eds De Kok L. J. Stulen I. Rennenberg H. Brunold C. Rauser W. (The Hague: SPB Academic Publishing) 295–313.

[B30] Fahey J. W. Zalcmann A. T. Talalay P. (2001). The chemical diversity and distribution of glucosinolates and isothiocyanates among plants. *Phytochemistry* 56 5–51 10.1016/S0031-9422(00)00316-2 11198818

[B31] Falk K. L. Tokuhisa J. G. Gershenzon J. (2007). The effect of sulfur nutrition on plant glucosinolate content: physiology and molecular mechanisms. *Plant Biol.* 9 573–581 10.1055/s-2007-965431 17853357

[B32] Fenwick G. R. Heaney R. K. Mullin W. J. (1983). Glucosinolates and their breakdown products in food and food plants. *Crit. Rev. Food Sci. Nutr.* 18 123–201 10.1080/10408398209527361 6337782

[B33] Ferreira R. M. Teixeira A. R. (1992). Sulfur starvation in Lemna leads to degradation of ribulose-bisphosphate carboxylase without plant death. *J. Biol. Chem.* 267 7253–7257. 1559969

[B34] Frerigmann H. Böttcher C. Baatout D. Gigolashvili T. (2012). Glucosinolates are produced in trichomes of *Arabidopsis thaliana*. *Front. Plant Sci.* 3:242 10.3389/fpls.2012.00242 PMC3483630 23115560

[B35] Gilbert S. Clarkson D. T. Cambridge M. Lambers H. Hawkesford M. J. (1997). Sulfate-deprivation has an early effect on the content of ribulose 1,5-bisphosphate carboxylase/oxygenase and photosynthesis in young leaves of wheat. *Plant Physiol.* 115 1231–1239 10.1104/pp.115.3.1231 12223869 PMC158588

[B36] Grubb C. D. Abel S. (2006). Glucosinolate metabolism and its control. *Trends Plant Sci.* 11 89–100 10.1016/j.tplants.2005.12.006 16406306

[B37] Gupta S. Sangha M. K. Kaur G. Atwal A. K. Kaur P. Kumar H. (2012). Biochem. *Anal. Biochem.* 1 121 10.4172/2161-1009.1000121

[B38] Halkier B. A. Du L. (1997). The biosynthesis of glucosinolates. *Trends Plant Sci.* 2 425–431 10.1016/S1360-1385(97)90026-1 20303821

[B39] Halkier B. A. Gershenzon J. (2006). Biology and biochemistry of glucosinolates. *Annu. Rev. Plant Biol.* 57 303–333 10.1146/annurev.arplant.57.032905.105228 16669764

[B40] Heaney R. K. Fenwick G. R. (1980). Glucosinolates in *Brassica* vegetables. analysis of 22 varieties of brussels sprouts (*Brassica oleracea* var. gemmifera). *J. Sci. Food Agr*. 31 785–793 10.1002/jsfa.2740310808

[B41] Hirai M. Y. Klein M. Fujikawa Y. Yano M. Goodenowe D. B. Yamazaki Y. (2005). Elucidation of gene-to-gene and metabolite-to-gene networks in *Arabidopsis* by integration of metabolomics and transcriptomics. *J. Biol. Chem.* 280 25590–25595 10.1074/jbc.M502332200 15866872

[B42] Hirai M. Y. Yano M. Goodenowe D. B. Kanaya S. Kimura T. Awazuhara M. (2004). Integration of transcriptomics and metabolomics for understanding of global responses to nutritional stresses in *Arabidopsis thaliana*. *Proc. Natl. Acad. Sci. U.S.A.* 101 10205–10210 10.1073/pnas.0403218101 15199185 PMC454188

[B43] Ishida M. Nagata M. Ohara T. Kakizaki T. Hatekeyama K. Nishio T. (2012). Small variation of glucosinolat composition in Japanese cultivars of radish (*Raphanus sativus* L.) requires simple quantitative analysis for breeding of glucosinolate component. *Breed.* *Sci.* 62 63–70 10.1270/jsbbs62.63 PMC3405960 23136515

[B44] Jones J. B. (1995). Determining total sulphur in plant tissue using the HACH kit spectrophotometer technique. *Sulphur* *Agric.* 19 58–62.

[B45] Kirkegaard J. A. Sarwar M. (1998). Biofumigation potential of Brassicas. *Plant Soil* 201 71–89 10.1023/A:1004364713152

[B46] Kissen R. Bones A. M. (2009). Nitrile-specifier proteins involved in glucosinolate hydrolysis in *Arabidopsis thaliana*. *J. Biol. Chem.* 284 12057–12070 10.1074/jbc.M807500200 19224919 PMC2673275

[B47] Kissen R. Rossiter J. Bones A. (2009). The ‘mustard oil bomb’: not so easy to assemble?! Localization, expression and distribution of the components of the myrosinase enzyme system. *Photochem. Rev.* 8 69–86 10.1007/s11101-008-9109-1

[B48] Kopriva S. Mugford S. J. Baraniecka P. Lee B. Matthewman C. A. Koprivova A. (2012). Control of sulfur partitioning between primary and secondary metabolism in *Arabidopsis*. *Front. Plant. Sci.* 3:163 10.3389/fpls.2012.00163 PMC3400089 22833750

[B49] Koralewska A. Stuiver C. E. E. Posthumus F. S. Kopriva S. Hawkesford M. J. De Kok L. J. (2008). Regulation of sulfate uptake, expression of the sulfate transporters Sultr1;1 and Sultr1;2, and APS reductase in Chinese cabbage (*Brassica pekinensis*) as affected by atmospheric H_2_S nutrition and sulfate deprivation. *Funct. Plant Biol.* 35 318–327 10.1071/FP07283 32688787

[B50] Kushad M. M. Brown A. F. Kurilich A. C. Juvik J. A. Klein B. P. Wallig M. A. (1999). Variation of glucosinolates in vegetable crops of *Brassica oleracea*. *J. Agric. Food Chem.* 47 1541–1548 10.1021/jf980985s 10564014

[B51] Maas F. M. Hoffmann I. Van Harmelen M. J. De Kok L. J. (1986). Refractometric determination of sulphate and other anions in plants separated by high-performance liquid chromatography. *Plant Soil* 91 129–132 10.1007/BF02181825

[B52] O’Callaghan K. J. Stone P. J. Hu X. Griffiths D. W. Michael R. Davey M. R. (2000). Effects of glucosinolates and flavonoids on colonization of the roots of *Brassica napus* by *Azorhizobium caulinodans* ORS571. *Appl. Environ. Microbiol.* 66 2185–2191 10.1128/AEM.66.5.2185-2191.2000 10788398 PMC101471

[B53] Rennenberg H. Herschbach C. (2014). A detailed view on sulphur metabolism at the cellular and whole-plant level illustrates challenges in metabolite flux analyses. *J. Exp. Biol.* 65 5711–5724 10.1093/jxb/eru315 25124317

[B54] Schiestl F. P. (2014). Correlation analyses between volatiles and glucosinolates show no evidence for chemical defense signaling in *Brassica rapa*. *Front. Ecol. Evol.* 2:10 10.3389/fevo.2014.00010

[B55] Schnug E. (1990). “Glucosinolates – fundamental, environmental and agricultural aspects,” in *Sulfur Nutrition and Sulfur Assimilation in Higher Plant; Fundamental, Environmental and Agricultural Aspects* eds Rennenberg H. Brunold C. De Kok L. J. Stulen I. (The Hague: SPB Academic Publishing), 97–106.

[B56] Schnug E. (1993). “Physiological functions and environmental relevance of sulfur-containing secondary metabolites,” in *Sulfur Nutrition and Sulfur Assimilation in Higher Plant; Regulatory Agricultural and Environmental Aspects* eds De Kok L. J. Stulen I. Rennenberg H. Brunold C. Rauser W. E. (The Hague: SPB Academic Publishing), 179–190.

[B57] Shahbaz M. Stuiver C. E. E. Posthumus F. S. Parmar S. Hawkesford M. J. De Kok L. G. (2014). Copper toxicity in Chinese cabbage is not influenced by plant sulphur status, but affects sulphur metabolism-related gene expression and the suggested regulatory metabolites. *Plant Biol.* 16 68–78 10.1111/plb.12019 23648043

[B58] Stulen I. De Kok L. J. (1993). “Whole plant regulation of sulfate uptake and metabolism – a theoretical approach and comparison with current ideas on regulation of nitrogen metabolism,” in *Sulfur Nutrition and Assimilation in Higher Plants; Regulatory, Agricultural and Environmental Aspects* eds De Kok L. J. Stulen I. Rennenberg H. Brunold C. Rauser W. E. (The Hague: SPB Academic Publishing) 77–91.

[B59] Tausz M. De Kok L. J. Stulen I. Grill D. (1996). Physiological responses of Norway spruce trees to elevated CO_2_ and SO_2_. *J. Plant Physiol.* 148 362–367 10.1016/S0176-1617(96)80266-5

[B60] Thies W. (1982). Complex-formation between glucosinolates and tetrachloropalladate (II) and its utilization in plant breeding. *Fette Seifen Anstrichmittel.* 84 388–342 10.1002/lipi.19820840903

[B61] Travers-Martin N. Kuhlmann F. Müller C. (2008). Revised determination of free and complexed myrosinase activities in plant extracts. *Plant Physiol. Biochem.* 46 506–516 10.1016/j.plaphy.2008.02.008 18395461

[B62] Van Dam N. M. Witjes L. Svatoš A. (2003). Interactions between aboveground and belowground induction of glucosinolates in two wild *Brassica* species. *New Phytol.* 161 801–810 10.1111/j.14698137.2004.00984.x 33873723

[B63] Verwoerd T. C. Dekker B. M. Hoekema A. (1989). A small-scale procedure for the rapid isolation of plant RNAs. *Nucleic Acids Res.* 17:2362 10.1093/nar/17.6.2362 PMC317610 2468132

[B64] Westerman S. Blake-Kalff M. A. A. De Kok L. J. Stuiver C. E. E. Stulen I. (2001a). Sulfate uptake and utilization by two varieties of *Brassica oleracea* with different sulfur need as affected by atmospheric H_2_S. *Phyton (B Aires)* 41 49–62.

[B65] Westerman S. Stulen I. Suter M. Brunold C. De Kok L. J. (2001b). Atmospheric H_2_S as sulfur source for *Brassica oleracea*: consequences for the activity of the enzymes of the assimilatory sulfate reduction pathway. *Plant Physiol. Biochem.* 39 425–432 10.1016/S0981-9428(01)01258-X

[B66] Westerman S. De Kok L. J. Stulen I. (2000a). Interaction between metabolism of atmospheric H_2_S in the shoot and sulfate uptake by the roots of curly kale (*Brassica oleracea* L). *Physiol. Plant.* 109 443–449 10.1034/j.1399-3054.2000.100411.x

[B67] Westerman S. Weidner W. De Kok L. J. Stulen I. (2000b). Effect of H_2_S exposure on 35S-sulfate uptake, transport and utilization in curly kale. *Phyton (B Aires)* 40 293–302.

[B68] Yang L. Stulen I. De Kok L. J. (2006a). Sulfur dioxide: relevance of toxic and nutritional effects for Chinese cabbage. *Environ. Exp. Bot.* 57 236–245 10.1016/j.envexpbot.2005.06.002

[B69] Yang L. Stulen I. De Kok L. J. (2006b). Impact of sulfate nutrition on the utilization of atmospheric SO_2_ as sulfur source for Chinese cabbage. *J. Plant Nutr. Soil Sci.* 169 529–534 10.1002/jpln.200520574

